# Identification of Conversion from Mild Cognitive Impairment to Alzheimer's Disease Using Multivariate Predictors

**DOI:** 10.1371/journal.pone.0021896

**Published:** 2011-07-21

**Authors:** Yue Cui, Bing Liu, Suhuai Luo, Xiantong Zhen, Ming Fan, Tao Liu, Wanlin Zhu, Mira Park, Tianzi Jiang, Jesse S. Jin, the Alzheimer's Disease Neuroimaging Initiative

**Affiliations:** 1 School of Design, Communication and Information Technology, University of Newcastle, Newcastle, New South Wales, Australia; 2 LIAMA Center for Computational Medicine, National Laboratory of Pattern Recognition, Institute of Automation, Chinese Academy of Sciences, Beijing, People's Republic of China; 3 Neuropsychiatric Institute, Prince of Wales Hospital, Randwick, Sydney, New South Wales, Australia; 4 Brain and Ageing Research Program, School of Psychiatry, University of New South Wales, Sydney, New South Wales, Australia; 5 Key Laboratory for NeuroInformation of Ministry of Education, School of Life Science and Technology, University of Electronic Science and Technology of China, Chengdu, People's Republic of China; Institute Biomedical Research August Pi Sunyer (IDIBAPS) - Hospital Clinic of Barcelona, Spain

## Abstract

Prediction of conversion from mild cognitive impairment (MCI) to Alzheimer's disease (AD) is of major interest in AD research. A large number of potential predictors have been proposed, with most investigations tending to examine one or a set of related predictors. In this study, we simultaneously examined multiple features from different modalities of data, including structural magnetic resonance imaging (MRI) morphometry, cerebrospinal fluid (CSF) biomarkers and neuropsychological and functional measures (NMs), to explore an optimal set of predictors of conversion from MCI to AD in an Alzheimer's Disease Neuroimaging Initiative (ADNI) cohort. After FreeSurfer-derived MRI feature extraction, CSF and NM feature collection, feature selection was employed to choose optimal subsets of features from each modality. Support vector machine (SVM) classifiers were then trained on normal control (NC) and AD participants. Testing was conducted on MCIc (MCI individuals who have converted to AD within 24 months) and MCInc (MCI individuals who have not converted to AD within 24 months) groups. Classification results demonstrated that NMs outperformed CSF and MRI features. The combination of selected NM, MRI and CSF features attained an accuracy of 67.13%, a sensitivity of 96.43%, a specificity of 48.28%, and an AUC (area under curve) of 0.796. Analysis of the predictive values of MCIc who converted at different follow-up evaluations showed that the predictive values were significantly different between individuals who converted within 12 months and after 12 months. This study establishes meaningful multivariate predictors composed of selected NM, MRI and CSF measures which may be useful and practical for clinical diagnosis.

## Introduction

Mild cognitive impairment (MCI) has been conceptualized as a disorder situated in the spectrum between normal cognition and dementia. However, only a proportion of individuals with MCI progress to dementia. Consequently, prediction of the likelihood of MCI individuals developing Alzheimer's disease (AD) is increasingly essential. Moreover, successful prediction offers the opportunity for the enrichment of clinical trials of disease-modifying therapies which aim to slow or prevent AD.

Presently, there are few clinical or imaging markers for the early identification of MCI which progresses to AD and MCI which does not progress. Based upon subsequent diagnosis status at follow-up evaluations, MCI participants can be divided into two subgroups: MCI patients who have converted to AD (MCI converters, MCIc), and MCI patients who have not converted to AD (MCI non-converters, MCInc). Different modalities of disease indicators have been studied for AD progression including neuroimaging biomarkers [1], [2], [3], [4], [5], biomedical biomarkers [6], and neuropsychological assessments [7], [8], [9]. Structural magnetic resonance imaging (MRI) captures disease-related structural patterns by measuring loss of brain volume and decreases in cortical thickness. A number of studies, covering region of interest (ROI), volume of interest, voxel-based morphometry and shape analysis, have reported that the degree of atrophy in several brain regions, such as the hippocampus, entorhinal cortex and medial temporal cortex, are sensitive to disease progression and predict MCI conversion [10], [11], [12], [13], [14], [15]. Biochemical changes in the brain are reflected in the cerebrospinal fluid (CSF). CSF concentrations of total tau (t-tau), amyloid-β 1 to 42 peptide (Aβ_1–42_) and tau phosphorylated at the threonine 181 (p-tau_181p_) are considered to be CSF biomarkers which are diagnostic for AD [6], [16], [17]. An increase in levels of CSF t-tau and a decline in Aβ_1–42_ have been identified as being amongst the most promising and informative AD biomarkers [6], [18]. Neuropsychological assessments are potentially useful for disease prognosis. Some cognitive measurements have shown statistically significant differences between MCI progressors and nonprogressors over the course of 12 months [19].

While most research focuses on a single modality of data, different modalities of data may provide complementary information. A recent study showed that a combination of MRI, CSF and fluorodeoxyglucose positron emission tomography (FDG-PET) predicted MCI converters within 18 months with a sensitivity of 91.5% and a specificity of 73.4% (total 99 individuals) [20]. Davatzikos and colleagues analyzed MRI and CSF biomarkers and correctly classified 55.8% (sensitivity, 94.7%; specificity, 37.8%) of 239 individuals as either MCIc or MCInc using SPARE-AD (Spatial Pattern of Abnormalities for Recognition of Early AD) index [15]. Ewers et al. [21] obtained accuracies from 64% to 68.5% for 130 MCI participants with different markers: MRI, CSF, neuropsychological tests, and their combinations.

Although significant progress has been made, most investigations concerning MCI prediction have chosen features based on prior knowledge and findings. To the best of our knowledge, few publications have selected the most relevant features automatically, thereby eliminating the scope for redundancy in MCI prediction. In this study, using an Alzheimer's Disease Neuroimaging Initiative (ADNI) dataset, we employed data-driven techniques and examined single and multiple modalities of features to capture MCI conversion within 24 months; we also analyzed conversion time. Firstly, structural measures of each ROI were extracted using FreeSurfer; CSF biomarkers and neuropsychological and functional measures (NMs) were downloaded from the ADNI website. Secondly, feature selection was performed on three modalities of features, respectively, in order to select optimal feature subsets. Finally, support vector machine (SVM) classifiers were trained to classify MCI individuals using selected features. Training was conducted on baseline normal control (NC) and AD groups, and testing was conducted on the baseline MCI group. Our hypothesis was that there could be symptoms of brain structural and functional deficits in the MCIc group, but not (much) in MCInc group, which could be identified at baseline. Previous research about spatial patterns of brain atrophy has demonstrated that characteristics of the MCIc group almost entirely overlap with those of AD individuals, and MCInc group characteristics almost entirely overlap with those of NC individuals [22]. Additionally, studies by Fan et al. [22], Costafreda et al. [10] and McEvoy et al. [13] successfully predicted MCIc using classifiers constructed from NC and AD participants, suggesting our hypothesis was convincing. Theoretically, classifiers constructed on MCI individuals should be able to separate MCIc/MCInc accurately; however, the follow-up of 24 months is not sufficient to obtain ground truth labels of MCIc/MCInc, which can only be achieved a much longer time-frame. In our study, some MCInc participants converted after 24 months, and the use of MCI participants for model generation may result in high training errors. For these reasons classifiers were constructed on NC and AD participants, and then applied to MCI individuals. We hypothesized that the combination of different modes of data would achieve better results because each modality separately produces a limited prediction. On the other hand, cross-sectional baseline differences between MCInc and MCIc would be most like NC and AD, respectively. In other words, the individuals with MCI who are about to develop AD would appear more similar to AD, whereas those who will not convert to AD would appear more similar to NC within selected features.

## Materials and Methods

### Ethics

For the purpose of this study we used ADNI data that were previously collected across 50 sites. Study subjects gave written informed consent at the time of enrollment for data collection and completed questionnaires approved by each participating site's Institutional Review Board (IRB), including Albany Medical College, Banner Alzheimer's Institute and Baylor College of Medicine etc. The complete list of ADNI sites' IRBs can be found at the link: http://adni.loni.ucla.edu/about/data-statistics/, or in Text S1.

### Participants

Data used in the preparation of this article were obtained from the ADNI database (www.loni.ucla.edu/ADNI) in April 2010. The ADNI was launched in 2003 by the National Institute on Aging, the National Institute of Biomedical Imaging and Bioengineering, the Food and Drug Administration, private pharmaceutical companies and non-profit organizations, as a $US60 million, 5-year public–private partnership. The primary goal of the ADNI has been to test whether serial MRI, positron emission tomography (PET), other biological markers, and cognitive and neuropsychological assessment can be combined to measure the progression of MCI and early AD. Determination of sensitive and specific markers of very early AD progression is intended to aid researchers and clinicians to develop new treatments and monitor their effectiveness, as well as lessen the time and cost of clinical trials. For up-to-date information, please refer to: http://www.adni-info.org.

The eligibility criteria for the inclusion of participants are described at: http://www.adni-info.org/Scientists/ADNIGrant/ProtocolSummary.aspx. General inclusion/exclusion criteria are as follows: normal subjects had Mini-Mental State Examination (MMSE) [23] scores between 24 and 30 (inclusive), a Clinical Dementia Rating (CDR) [24] of 0, and were non depressed, non MCI, and non demented. MCI patients had MMSE scores between 24 and 30 (inclusive), a memory complaint, had objective memory loss measured by education adjusted scores on the Wechsler Memory Scale Logical Memory II [25], a CDR of 0.5, absence of significant levels of impairment in other cognitive domains, essentially preserved activities of daily living, and an absence of dementia. AD patients had MMSE scores between 20 and 26 (inclusive), a CDR of 0.5 or 1.0, and met NINCDS/ADRDA [26] criteria for probable AD.

Only ADNI subjects who had pre-processed and quality checked MR images, baseline CSF measurements and at least 24-month follow-up evaluations were included in this study. This yielded a total of 87 MCInc, 56 MCIc, 111 NC and 96 AD patients. Table 1 provides detailed participant demographics information for training data and test data. There were no significant differences between NC and AD, MCInc and MCIc groups in terms of age and sex. We focused on baseline classification of MCI individuals, therefore MRI scans, CSF biomarkers, demographic information and neuropsychological data were all obtained at the baseline visit.

**Table 1:** Participant demographic characteristics.

	Training data			Test data		
	NC (n = 111)	AD (n = 96)	*p* a	MCInc (n = 87)	MCIc (n = 56)	*p* b
**Age**	75.4±5.12	74.8±8.01	0.454	74.3±6.98	75.02±7.49	0.585
**Male (%)**	50.5	58.3	0.256	63.2	67.9	0.570
**Education (years)**	15.7±2.81	15.2±3.35	0.196	16.4±2.74	15.4±3.11	0.042
**ApoE ε4 carriers (%)**	23.4	69.8	<0.001	47.1	64.3	0.045
**MMSE**	29.07±1.0	23.51±1.9	<0.001	27.13±1.7	26.57±1.9	0.066
**CDR**	0±0	0.7±0.25	<0.001	0.5±0	0.5±0	——

Note: Values are mean ± SD unless otherwise indicated. NC, normal control; AD, Alzheimer's Disease; MCInc, mild cognitive impairment patients who have not converted to AD within 24 months; MCIc, mild cognitive impairment patients who have converted to AD within 24 months; ApoE, apolipoprotein E; MMSE, Mini-Mental State Examination; CDR, Clinical Dementia Rating.

a Two sample *t*-test for all comparisons between NC and AD groups except sex and ApoE ε4 carriers, where Pearson's chi-square tests were used.

b Two sample *t*-test for all comparisons between MCInc and MCIc groups except sex and ApoE ε4 carriers, where Pearson's chi-square tests were used.

### MRI imaging acquisition

Structural MRI scans were acquired from 1.5T scanners at multiple sites across the United States and Canada. MRI protocols ensured comparability across a variety of scanners (GE, Siemens or Philips). The imaging sequence was a 3-dimentional sagittal magnetization prepared rapid gradient-echo (MPRAGE). The MPRAGE sequence was repeated back-to-back to increase the likelihood of acquiring at least one good quality MPRAGE scan. In addition, a dual fast spin-echo (proton density/T2-weighted) sequence was acquired to evaluate the presence or state of vascular disease and general pathology detection [27], [28]. The pre-processing correction procedure was as follows: (1) grad warp correction of image geometry distortion due to gradient non-linearity; (2) B1 non-uniformity processing to correct the image intensity non-uniformity; and (3) N3 processing to reduce residual intensity non-uniformity [28]. Original scans and pre-processed images are available at http://adni.loni.ucla.edu/.

### Overview of prediction procedure

**Figure 1:**
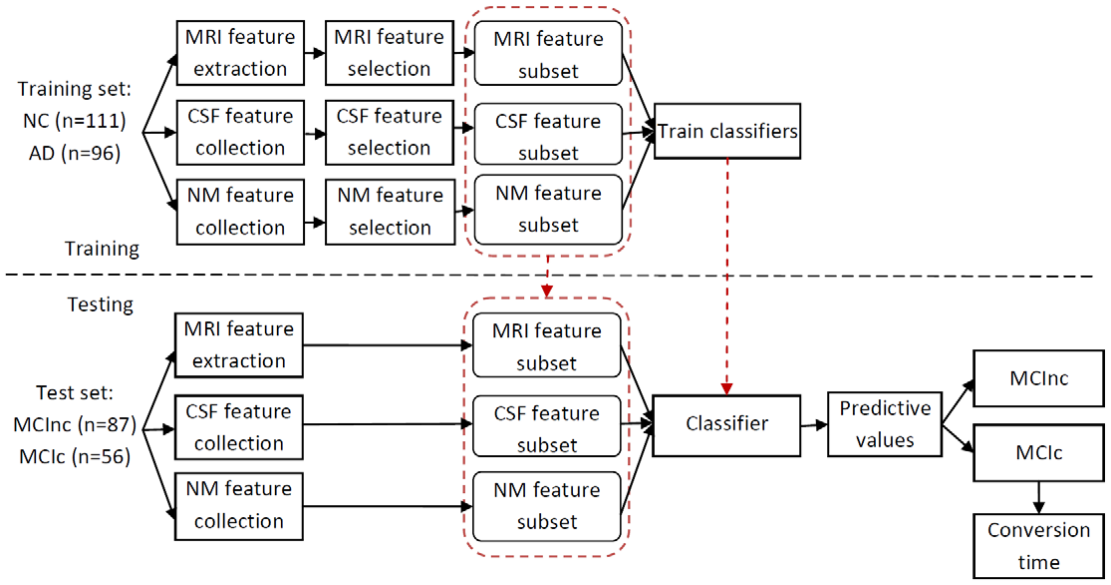
Overview of the prediction procedure.

The prediction procedure consisted of three processing stages: feature extraction and collection, optimal feature subset selection, and classification. Figure 1 illustrates the diagram of the prediction framework. During the training stage, MRI features which had been extracted automatically using FreeSurfer, as well as a set of NM and CSF biomarkers, were downloaded from the ADNI website. A feature selection method was then employed to choose optimal subsets of features, respectively. After feature selection, we combined multiple features, including the MRI, NM and CSF features to train classifiers to distinguish between NC and AD. In the testing stage, we extracted what we had determined to be the optimal feature subsets during the training stage. A predictive value was then generated for each test subject through the SVM classifier.

### MRI feature extraction

Advances in MR image analysis algorithms have led to the development of automated parcellation tools which can segment the whole brain into anatomic regions and quantify the features of each region [29]. The widely used FreeSurfer software package (http://surfer.nmr.mgh.harvard.edu/) was applied to each participant's pre-processed scan. Processing results using FreeSurfer Version 4.3.0 have been published on the website: www.loni.ucla.edu/ADNI. Briefly, the processing included automated Talairach space transformation, intensity inhomogeneity correction, removal of non-brain tissues, intensity normalization, tissue segmentation (the subcortical structures, brain stem, cerebellum, and cerebral cortex) [30], [31], automated correction of topology defects, surface deformation to form the gray/white matter boundary and gray matter/CSF boundary [32], and parcellation of the cerebral cortex [33]. The atlas used, detailed in [33], included 34 cortical ROIs per hemisphere. For each ROI, the cortical thickness average (TA), standard deviation of thickness (TS), surface area (SA) and cortical volume (CV) were calculated as features. SA was calculated as the area of the surface layer equidistant between the gray/white matter and gray matter/CSF surfaces. CV at each vertex over the whole cortex was computed by the product of the SA and thickness at each surface vertex. Left and right hemisphere SA and total intracranial volume (ICV) were also included. For each subcortical structure, the subcortical volume (SV) was extracted. This yielded a total of 323 MRI features including 279 cortical and 44 subcortical features (see Table S1).

### CSF biomarker collection

Baseline CSF samples were obtained through lumbar puncture at all participating sites. The CSF collection and transportation protocols and details on CSF are described in [6] and on the ADNI website (http://www.adni-info.org/Scientists/ADNIScientistsHome.aspx). CSF concentrations of t-tau, Aβ_1–42_ and p-tau_181p_ were measured, as were ratios of t-tau to Aβ_1–42_, and p-tau_181p_ to Aβ_1–42_. CSF features for subjects taken at baseline are listed in Table 2.

**Table 2:** Baseline CSF biomarker concentrations and ratios of subjects.

	t-tau(pg/ml)	Aβ_1–42_ (pg/ml)	p-tau_181p_ (pg/ml)	t-tau/Aβ_1–42_	p-tau_181p_/Aβ_1–42_
**NC (n = 111)**	69.8±30.6	205.6±55.6	24.9±14.6	0.39±0.3	0.14±0.1
**AD (n = 96)**	122.9±58.0	142.8±40.0	42.2±20.1	0.93±0.5	0.32±0.2
**MCInc (n = 87)**	96.1±53.2	163.6±58.5	34.3±17.1	0.72±0.6	0.26±0.2
**MCIc (n = 56)**	110.5±45.1	142.2±35.9	39.5±15.5	0.82±0.3	0.30±0.1

Note: Values are mean ± SD. NC, normal control; AD, Alzheimer's Disease; MCInc, mild cognitive impairment patients who have not converted to AD within 24 months; MCIc, mild cognitive impairment patients who have converted to AD within 24 months; CSF, cerebrospinal fluid; t-tau, total tau; p-tau_181p_, tau phosphorylated at the threonine 181; Aβ_1–42,_ amyloid-β 1 to 42 peptide.

### NM collection

NMs were undertaken at the time of scan acquisition as shown in Table 3. Neuropsychological tests used in this study include Logical Memory II (LM) [25], Auditory Verbal Learning Test (AVLT) [34], category fluency and digit span, Trail Making Tests A and B [35], Boston Naming Test [36], and clock drawing [37]. The Functional Assessment Questionnaire (FAQ) [38] was used for functional testing. Details of ADNI NMs are available at (http://adni.loni.ucla.edu/wp-content/uploads/2010/09/BLCogTestingWorksheet.pdf) and on the ADNI cognitive testing webpage: http://www.adni-info.org/Scientists/CognitiveTesting.aspx.

**Table 3:** Baseline neuropsychological and functional measures.

Assortment variable	NC (n = 111)	AD (n = 96)	MCInc (n = 87)	MCIc (n = 56)
LM delayed recall	12.6±3.6	1.1±1.8	4.3±2.7	2.8±2.3
LM immediate recall	13.5±3.6	3.9±2.9	7.5±2.5	6.0±3.0
Boston Naming Test	27.5±2.4	23.1±6.1	25.8±4.1	25.7±3.7
AVLT trials 1–5	43.3±8.4	23.4±7.1	32.8±8.9	26.6±6.7
AVLT delayed recall	7.3±3.5	1±2.0	3.3±3.4	1.5±1.9
AVLT delayed recall/trial 5 (%)	66.5±30.0	14.2±25.0	35.2±30.6	21.1±26.7
Category fluency (vegetable)	14.4±3.8	8.1±3.4	11.1±3.5	10.1±3.1
Category fluency (animal)	19.3±5.7	12.8±4.9	16.3±4.8	15.6±4.8
Trail Making Test A, s	36.7±13.5	68.4±38.7	42.6±20.6	49.6±27.2
Trail Making Test B, s	88.5±41.5	204.3±86.7	118.2±63.2	144.6±71.2
Clock drawing	4.6±0.7	3.3±1.3	4.3±0.9	3.8±1.2
Digit forwards	8.7±2.1	7.8±1.9	8.1±2.1	8.5±1.9
Digit backwards	7.0±2.2	4.9±1.8	6.3±2.0	6.2±1.7
FAQ	0.2±0.7	12.7±6.7	2.8±3.9	5.2±4.4

Note: Values are mean ± SD. NC, normal control; AD, Alzheimer's Disease; MCInc, mild cognitive impairment patients who have not converted to AD within 24 months; MCIc, mild cognitive impairment patients who have converted to AD within 24 months; LM, logical memory II; AVLT, Auditory Verbal Learning Test; FAQ, Functional Assessment Questionnaire.

### Feature selection

Of the pool of available features, some were sensitive and relevant to AD and some were less relevant or redundant for classification. We therefore performed a feature selection procedure in NC and AD groups in order to identify the most characteristic structural AD-like patterns which could be looked for in MCInc and MCIc individuals. The approach applied for MRI and CSF features is a filter followed by a wrapper method, while we used a filter for NM feature selection.

#### MRI and CSF feature selection

An optimal feature subset is achieved by selecting the most relevant features and eliminating redundant features. Feature ranking followed by a wrapper method is accepted as a recommended part of a feature selection procedure [39]. Feature ranking evaluates all of the features by looking at the intrinsic characteristics of the data with respect to clinical evaluations. Wrapper methods evaluate the effectiveness of a subset by the accuracy (or AUC) of its classification. We performed the same feature selection approach for MRI and CSF features. During the feature ranking stage, we first linearly normalized all the features to the range between 0 and 1, since features have different scales. We then employed the minimum redundancy and maximum relevance (mRMR) filter method introduced by Peng et al. [40], [41]. This method computes the mutual information of two variables by their probabilistic density function. The mRMR feature ranking is obtained by optimizing two criteria, i.e., maximum relevance and minimum redundancy, simultaneously. The detailed implementation algorithm is described in [40], [41]. In order to select the optimal feature subset after feature ranking, we employed the popular classifier SVM by incrementally adding features based on their ranking (highest to lowest). Optimal features were selected when the highest AUC was obtained. We performed 10-fold cross-validation and repeated the procedure 20 times with training samples in order to identify robust and stable discriminative features. Selection frequency was computed by dividing the number of selection by the total number of times the procedure was repeated. The higher the selection frequency, the more stable and reliable the feature is for discrimination. In order to identify the most discriminative feature subset, we selected features with over 50% selection frequency. This yielded a subset of 7 features out of a possible 323 MRI features, and a subset of 2 features out of 5 CSF biomarkers.

#### NM feature selection

Our neuropsychological feature selection was performed using a filter method. A wrapper was not involved because NMs are very separable between NC and AD groups. If a wrapper were to be used, the highest accuracy would be achieved when using the top ranked feature. Therefore, only one feature can be selected in NC and AD groups, whereas this feature may be not an optimal subset for MCI classification. Therefore we filtered neuropsychological features based on two rankings: the maximal relevance method which ranked features based on mutual information between each feature and corresponding clinical labels [41], and the AUC values in SVM classification of each individual NM to discriminate between NC and AD. Note that linear feature normalization was applied before ranking. In order to reduce variability, we carried out two feature ranking schemes 20 times using 10-fold cross-validation on the training set.

### Classification using SVM

SVM is a powerful, supervised, classification algorithm for pattern classification that uses a kernel function to construct linear classification boundaries in high (often infinite) dimensional spaces [42]. It is widely accepted as one of the most powerful classifiers available. In SVM, the output in a linearly separable case has the form
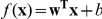
where **x** is an input vector.

For a given hyperplane (decision surface) described with the equation 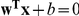, and for a vector **z** that does not belong to the hyperplane, the following is satisfied [42], [43]:
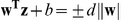
where *d* is the “distance” of the “point” **z** to the given hyperplane. Therefore the output *f(x)* (i.e. predictive value) of the SVM is actually proportional to the norm of vector **w** and the distance *d(x)* from the chosen hyperplane. In a non-linear case, we still look for a linear separation hyperplane within the mapped feature space. For each MCI participant, the classifier generated a continuous predictive value, which was then forced to be either positive (MCIc) or negative (MCInc) using threshold decision rules. The relationship between predictive values and conversion time was then analyzed. In the present study, SVM classifiers were implemented using the LIBSVM toolbox [44] with the Gaussian radial basis function (RBF) kernel, i.e. 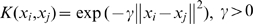. Unlike the linear kernel, the RBF kernel can handle cases where the relationship between clinical labels and features are nonlinear [45]. The parameters, C (a constant determining the tradeoff between training error and model flatness) and 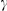 (Gaussian kernel width) were optimized via cross-validation on the training data. Note that, as different features had different scales, we linearly scaled each training feature to conform to a range between 0 and 1; the same scaling method was subsequently applied to the test data.

## Results

### Discriminating MRI, CSF and NM features

Optimal MRI and CSF feature subsets are summarized in Table 4 and Table 5. For selected MRI features, the subcortical region was the hippocampus and the cortical regions included the entorhinal cortex, middle temporal gyrus, inferior parietal cortex and retrosplenial cortex. The thickness of the left entorhinal cortex was the highest ranked with 91.50% selection frequency. The volume of the right middle temporal gyrus was ranked second with 88.50% selection frequency. Volumes of the right and left hippocampus were also important features ranking third and fourth, respectively, followed by the thickness of the right inferior parietal cortex, left retrosplenial cortex and left middle temporal gyrus. *t*-tests of the 7 features showed statistically significant differences between NC and AD groups. Meanwhile, *t*-tests of MCInc and MCIc groups showed significant differences, with the exception of average thickness of the left entorhinal cortex and retrosplenial cortex. For CSF features, t-tau/Aβ_1–42_ and p-tau_181p_/Aβ_1–42_ were selected. There were significant differences between NC and AD subjects, but no significant differences found between MCInc and MCIc individuals (see Table 5).

**Table 4:** Selected MRI features.

Ranking	Selection frequency (%)	Feature	Type	NC vs. AD		MCInc vs. MCIc	
				*p*	Corr. *p* a	*p*	Corr. *p* a
1	91.50	Entorhinal Cortex L	TA	<0.0001	**<0.0001**	0.1735	1.0
2	88.50	Middle Temporal Gyrus R	CV	<0.0001	**<0.0001**	0.0008	**0.0056**
3	71.00	Hippocampus R	SV	<0.0001	**<0.0001**	0.0015	**0.0105**
4	65.50	Hippocampus L	SV	<0.0001	**<0.0001**	0.0063	**0.0441**
5	60.00	Inferior Parietal Cortex R	TA	<0.0001	**<0.0001**	0.0008	**0.0056**
6	59.00	Retrosplenial Cortex L	TA	<0.0001	**<0.0001**	0.0222	0.1554
7	53.00	Middle Temporal Gyrus L	TA	<0.0001	**<0.0001**	0.0012	**0.0084**

Note: NC, normal control; AD, Alzheimer's disease; MCInc, mild cognitive impairment patients who have not converted to AD within 24 months; MCIc, mild cognitive impairment patients who have converted to AD within 24 months; CV, cortical volume; TA, cortical thickness average; SV, subcortical volume; L, left hemisphere; R, right hemisphere.

a Bonferroni-corrected (Corr.) *p* values are shown after controlling for multiple comparisons, with significant differences in bold (Corr. *p*<0.05).

**Table 5:** Selected CSF features.

Ranking	Selection frequency (%)	Feature	NC vs. AD		MCInc vs. MCIc	
			*p*	Corr. *p* a	*p*	Corr. *p* a
1	94.5%	t-tau/Aβ_1–42_	<0.0001	**<0.0001**	0.2894	0.5788
2	56.5%	p-tau_181p_/Aβ_1–42_	<0.0001	**<0.0001**	0.1351	0.2702

Note: NC, normal control; AD, Alzheimer's disease; MCInc, mild cognitive impairment patients who have not converted to AD within 24 months; MCIc, mild cognitive impairment patients who have converted to AD within 24 months; t-tau, total tau; Aβ_1–42_, amyloid-β 1 to 42 peptide; p-tau_181p_, tau phosphorylated at the threonine 181.

a Bonferroni-corrected (Corr.) *p* values are shown after controlling for multiple comparisons, with significant differences in bold (Corr. *p*<0.05).

The rankings of 14 NM features based on two schemes are presented in Table 6. We chose measures with a correlation coefficient above 0.3 and classification AUC above 0.95 in order to select the most discriminate features. 5 NM features were selected, including FAQ, LM delayed recall, LM immediate recall, AVLT delayed recall and AVLT trials 1–5 (see Table 7). Statistical analysis showed all of the selected features to be significantly different between NC and AD, and between MCInc and MCIc groups.

**Table 6:** Neuropsychological feature ranking.

Ranking 1	Correlation coefficient	Ranking 2	Classification AUC	Neuropsychological and functional test
1	0.4832	2	0.9889	FAQ
2	0.4742	1	0.9990	LM delayed recall
3	0.3832	3	0.9794	LM immediate recall
4	0.3219	5	0.9576	AVLT delayed recall
5	0.3160	4	0.9685	AVLT trials 1–5
6	0.2741	6	0.9211	AVLT delayed recall/trial 5
7	0.2594	9	0.8677	Trail Making Test B
8	0.2392	7	0.9068	Category fluency (vegetable)
9	0.1973	12	0.8015	Trail Making Test A
10	0.1917	8	0.8976	Clock drawing
11	0.1560	10	0.8275	Category fluency (animal)
12	0.1176	13	0.7730	Boston Naming Test
13	0.1074	11	0.8236	Digit backwards
14	0.0384	14	0.6588	Digit forwards

Note: FAQ, Functional Assessment Questionnaire; LM, logical memory II; AVLT, Auditory Verbal Learning Test.

**Table 7:** Selected NM features.

Ranking	Feature	NC vs. AD		MCInc vs. MCIc	
		*p*	Corr. *p* a	*p*	Corr. *p* a
1	FAQ	<0.0001	**<0.0001**	0.00082	**0.0041**
2	LM delayed recall	<0.0001	**<0.0001**	0.00078	**0.0039**
3	LM immediate recall	<0.0001	**<0.0001**	0.00188	**0.0094**
4	AVLT delayed recall	<0.0001	**<0.0001**	0.00031	**0.0016**
5	AVLT trials 1–5	<0.0001	**<0.0001**	0.00002	**0.0001**

Note: NC, normal control; AD, Alzheimer's disease; MCInc, mild cognitive impairment patients who have not converted to AD within 24 months; MCIc, mild cognitive impairment patients who have converted to AD within 24 months; NM, neuropsychological and functional measure; LM, logical memory II; AVLT, Auditory Verbal Learning Test; FAQ, Functional Assessment Questionnaire.

a Bonferroni-corrected (Corr.) *p* values are shown after controlling for multiple comparisons, with significant differences in bold (Corr. *p*<0.05).

### Classification performance using single and multiple modalities of features

We trained SVM classifiers using selected NM, MRI and CSF measures to discriminate between NC and AD participants, and tested on MCI participants. As shown in Table 8, NM method achieved a good AUC (0.761), for which it outperforms individual MRI (0.650) and CSF (0.641) method. Combining NM and CSF/MRI features increased the classification performance. The best performance was achieved using a combination of three modalities of features, i.e., NM, CSF and MRI, which had an accuracy of 67.13%, a sensitivity of 96.43%, a specificity of 48.28%, and an AUC of 0.796.

**Table 8:** Classification of MCIc versus MCInc at baseline.

Method	Accuracy (%)	Sensitivity (%)	Specificity (%)	AUC
**NM, CSF, MRI**	67.13	96.43	48.28	0.796
**NM, CSF**	65.04	94.64	45.98	0.784
**NM, MRI**	62.24	92.86	42.53	0.781
**NM**	65.04	91.07	48.28	0.761
**MRI, CSF**	58.74	71.43	50.57	0.673
**MRI**	62.24	57.14	65.52	0.650
**CSF**	60.84	80.36	48.28	0.641

Note: AUC, area under the receiver operating characteristic curve; MRI represents 7 selected structural features; NM represent 5 selected neuropsychological and functional measures; CSF represents 2 selected CSF features.

**Figure 2:**
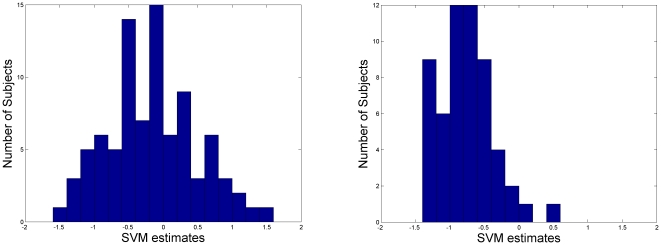
The histograms of SVM predictive values of MCInc (left) and MCIc (right).

**Figure 3:**
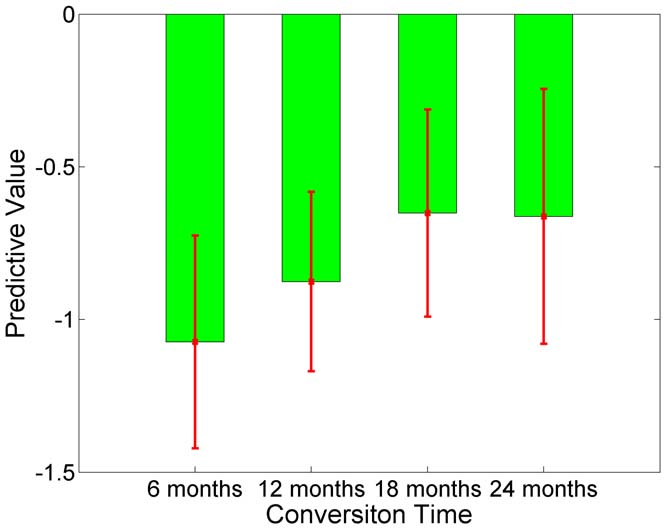
Predictive values of MCIc at different conversion time. Predictive values of MCIc at 6-month (−1.07±0.35), 12-month (−0.88±0.29), 18-month (−0.65±0.34) and 24-month (−0.66±0.42) follow-up evaluations.

**Figure 4:**
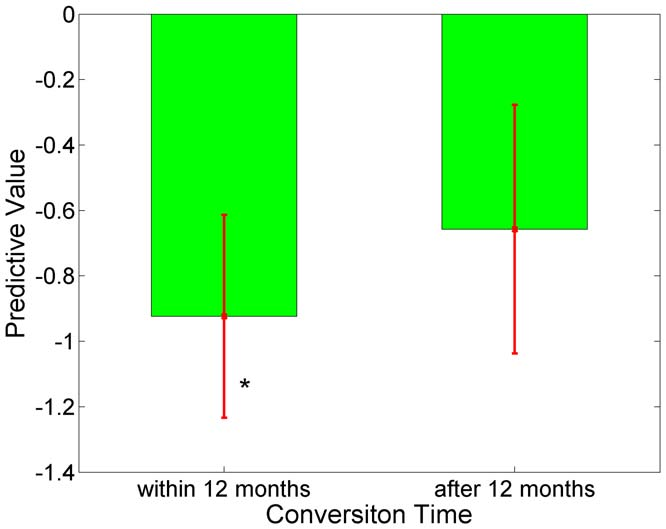
Predictive values of MCIc at different conversion time. Predictive values of MCIc within 12-month (−0.92±0.31) and after 12-month (−0.66±0.38) follow-up evaluations. *Significant differences between predictive values of conversion time within 12 months and after 12 months (*p*<0.01).

During the testing stage, the classifier generated a predictive value for each subject. Most MCIc subjects have negative predictive values which indicated the majority had been classified correctly (96.43%); while MCInc subjects have a wider range of predictive values from negative to positive values (see Figure 2). Further analysis of the predictive values at different conversion times using selected NM, MRI and CSF features is presented in Figure 3 and Figure 4. Specifically, MCIc subjects who converted at 6 months, 12 months, 18 months and 24 months are −1.07±0.35, −0.88±0.29, −0.65±0.34, and −0.66±0.42, respectively. Predictive values of MCIc subjects who converted within 12 months and after 12 months (before 24 months) are −0.92±0.31 and −0.66±0.38, respectively, which were significantly different (p<0.01, Figure 4).

## Discussion

Te present study examined the capability of single and multiple modalities of predictors to identify conversion from MCI to AD using pattern classification techniques. We used a feature selection approach and selected optimal feature subsets from different modalities. In addition, prediction of conversion time was investigated through the predictive values at different conversion times.

### Single mode predictors

Feature selection from MRI features provided a subset of discriminating structural measures. Our data-driven method showed that spatial atrophy predictors of MCI conversion included ROIs of the entorhinal cortex, inferior parietal cortex, retrosplenial cortex, middle temporal gyrus and hippocampus. Our results correspond with a number of previous studies showing that atrophy in these structures has been found to be predictive during disease progression. [1], [21], [29], [46], [47], [48]. These ROIs are from the episodic memory network and they served as the strongest predictors of memory performance, reflecting the association between regional atrophy and loss of memory [49], [50]. The entorhinal cortex and hippocampus atrophies are established imaging AD biomarkers [51]; both contribute to prediction [21], [47], [48]. Moreover, the entorhinal cortex and inferior parietal lobule are important predictors of time to progression [46]. In addition, we found the entorhinal cortex was the highest ranked of other morphometry features, even superior to hippocampus volumes. This is consistent with findings from previous studies [46], [47], [48]. t-tau/Aβ_1–42_ and p-tau_181p_/Aβ_1–42_ are the most sensitive predictors in the early diagnosis of AD. They both increase sensitivity in prognosis. Some studies [6], [52] have reported similar results.

Statistically significant differences between NC and AD groups illustrate that the selected MRI and CSF features were highly discriminative. The t-test conducted on evaluation results from MCInc and MCIc subjects showed that most features were statistically significant. This suggests that the trends involving features which discriminate between NC and AD may also distinguish between MCInc and MCIc subjects. Although the entorhinal cortex, t-tau/Aβ_1–42_ and p-tau_181p_/Aβ_1–42_ were not significantly different, our results indicated that they were indispensable since the combination of features performed better, suggesting that these features are mutually complementary and that their combination works as a good classificatory predictor. An additional factor concerned short-term follow-ups. These influence labels of MCInc and we found that subjects changed from MCInc to MCIc when evaluations were provided over a longer period of time.

Neuropsychological tests are strong descriptors for the decline of cognition from MCI to AD [7], [8]. Neuropsychological measures (either alone or combined with other predictors) are being widely investigated to predict which individuals progress to AD and which do not [7], [8], [21], [53]. MCIc/MCInc were labelled by both baseline and follow-up diagnoses, which required clinical examination and comprehensive neuropsychological assessments, therefore NM could be biased compared with MRI and CSF measures. Our results also indicated that NM achieved better prediction performance. Our findings of NM predictors included 5 features, which are significantly different between NC and AD groups, and between MCInc and MCIc groups. Classification performance for the use of all 14 NM features was comparable with the use of 5 selected features, suggesting our approach with feature selection is effective since simple and relatively fewer markers might make prediction more practical. We found that LM delayed recall was especially sensitive in distinguishing between MCInc and MCIc groups. This is consistent with related research which has shown that this test has typically greater power (highest loading) in predicting conversion to AD [7], [9], [54]. While relatively few studies have included functional measures in the detection of MCI conversion, our findings indicated that inclusion of FAQ scores was important for achieving a sensitive indicator of disease progression.

### Multivariate predictors

NMs outperformed FreeSurfer-derived MRI and CSF features and attained a good AUC. However, multimodal feature combination appears more promising. The combination of NM, MRI and CSF features outperformed any single modality of data. The high sensitivity suggests this combination may be a good predictor for prognosis of MCI. Our results marginally outperformed Davatzikos et al.'s state-of-the-art study [15] in terms of accuracy, sensitivity, specificity and AUC. Our results are consistent with their findings that MCIc had mostly AD-like baseline markers, while MCInc had mixed markers, suggesting that some MCInc participants may convert later [15]. For example, in our study, 10 MCInc subjects had 36-month follow-ups. We found our classifier was able to detect 8 (80%) of them as converters, suggesting longer follow-up will clarify the specificity of baseline measures. We note that our accuracy is higher than Zhang et al.'s study, which used the combination of MRI, CSF and PET data [20], although our specificity is lower. In terms of accuracy, our method is comparable to Ewers et al.'s study, which used logistic regression and picked up features by prior knowledge [21]. It is problematic to compare classification results from studies using different populations, therefore we only compared our results with the studies using ADNI cohorts.

Taken together, MRI measures offer information regarding the structural degeneration of AD, CSF biomedical levels correspond with the pathological changes at the biological level, and NMs reflect the memory deficits and behavioral symptoms of AD. Of the three modalities of data, NMs are the most distinguishing, and MRI and CSF data provide complementary predictive information, which enhanced prediction performance and prognostic power overall. The optimal combination of these multimodal features would therefore enable greater insight into the disease, as they provide complementary information about AD progression.

While it is challenging to predict conversion time, it is highly significant for clinical diagnosis. In our study, MCI converters who converted within 12 months of follow-up have AD-like patterns; hence their predictive values are lower. Predictive values for MCI subjects who converted after 12 months are generally higher. Therefore our methodology appears to be a useful means for predicting conversion time.

### Limitations

Our study has some limitations. Firstly, we did not use weightings for different modalities when we combined them. Zhang et al.'s approach of using different weightings may improve the prediction performance of our method [20]. Another limitation is the relatively short interval of 24-month follow-up. A longer follow-up interval for MCInc subjects would make the ground truth labels more reliable because some MCI subjects may convert later. Accordingly, prediction specificity and accuracy could be better validated.

### Conclusions

The present study proposed multivariate predictors for tracking AD progression using pattern classification techniques. Multimodal features were combined after feature selection from structural MRI, CSF and NM measures. Classification results verify our hypothesis that the combination of multimodal features, including NM, MRI and CSF, outperforms a single modality of features, possibly because different features are mutually complementary. Our proposed multivariate predictors achieved good baseline accuracy and high sensitivity. In addition, predictive values of MCIc within 12 months and after 12 months are significantly different. Furthermore, the selected features have proved to be closely related to AD progression, which corresponds with the findings of recent studies and verifies the effectiveness of our feature selection method. In summary, our prediction procedure may be practical and helpful for clinical diagnosis.

## Supporting Information

Text S1 Ethics.
(DOC)

Table S1 FreeSurfer-derived MRI features.
(DOC)
