# Supplementary material for: Identification of Conversion from Mild Cognitive Impairment to Alzheimer's Disease Using Multivariate Predictors
Source: PLoS One. 2011 Jul 21;6(7):e21896. doi: 10.1371/journal.pone.0021896 (PMC3140993; doi:10.1371/journal.pone.0021896)
Supplement: Text S1 — Ethics. (DOC) [file pone.0021896.s001.doc]

**Ethics**

For the purpose of this study we used the Alzheimer’s Disease Neuroimaging Initiative (ADNI) data that were previously collected across 50 sites. Study subjects gave written informed consent at the time of enrollment for data collection and completed questionnaires approved by each participating site’s Institutional Review Board (IRB). The complete list of ADNI sites' IRBs can be found in the link: [http://adni.loni.ucla.edu/about/data-statistics/](https://ch1prd0202.outlook.com/owa/redir.aspx?C=a0e3aa713dca48d78560ffb044a05fd1&URL=http%3A%2F%2Fadni.loni.ucla.edu%2Fabout%2Fdata-statistics%2F). Specifically, they are: Albany Medical College, Banner Alzheimer’s Institute, Baylor College of Medicine, Boston University, Brigham and Women’s Hospital, Butler Hospital Memory & Aging Program, Case Western Reserve University, Cleveland Clinic, Columbia University, Darthmouth – Hitchcock Medical Center, Dent Neurologic Institute, Duke University Medical Center, Emory University, Georgetown University, Howard University, Indiana University, Jefferson Hospital for Neuroscience, Johns Hopkins University, Mayo Clinic, Jacksonville, Mayo Clinic, Rochester, McGill University/Jewish General Hospital Memory Clinic, Medical University of South Carolina, Mount Sinai School of Medicine, Neurological Care of Central New York, New York University Medical Center, Northwestern University, Ohio State University, Olin Neuropsychiatry Research Center, Oregon Health and Science University, Parkwood Hospital, Premiere Research Institute, Rhode Island Hospital, Rush University Medical Center, Saint Joseph’s Health Center, London, Ontario, Stanford University, Banner Sun Health Research Institute, Sunnybrook Health Sciences, University of Alabama, Birmingham, University of British Columbia, University of California, Davis, University of California, Irvine, University of California, Irvine-BIC, University of California, Los Angeles, University of California, San Diego, University of California, San Francisco, University of Kansas, University of Kentucky, University of Michigan, Ann Arbor, University of Nevada School of Medicine, Las Vegas, University of Pennsylvania, University of Pittsburgh, University of Rochester, University of Southern California, University of Texas Southwestern Medical Center, University of Wisconsin, Wake Forest University, Washington University St. Louis, Wein Center for Clinical Research and Yale University School of Medicine.
