# Supplementary material for: Identification of Conversion from Mild Cognitive Impairment to Alzheimer's Disease Using Multivariate Predictors
Source: PLoS One. 2011 Jul 21;6(7):e21896. doi: 10.1371/journal.pone.0021896 (PMC3140993; doi:10.1371/journal.pone.0021896)
Supplement: Table S1 — FreeSurfer-derived MRI features. (DOC) [file pone.0021896.s002.doc]

**Table S1** FreeSurfer-derived MRI features.

|  | **ROI** | **Laterality** | **Type** |
| --- | --- | --- | --- |
| Cortical features (n = 279) | Banks Superior Temporal Sulcus | L, R | CV, SA, TA, TS |
|  | Caudal Anterior Cingulate Cortex | L, R | CV, SA, TA, TS |
|  | Caudal Middle Frontal Gyrus | L, R | CV, SA, TA, TS |
|  | Cuneus Cortex | L, R | CV, SA, TA, TS |
|  | Entorhinal Cortex | L, R | CV, SA, TA, TS |
|  | Frontal Pole | L, R | CV, SA, TA, TS |
|  | Fusiform Gyrus | L, R | CV, SA, TA, TS |
|  | Inferior Parietal Cortex | L, R | CV, SA, TA, TS |
|  | Inferior Temporal Gyrus | L, R | CV, SA, TA, TS |
|  | Insula | L, R | CV, SA, TA, TS |
|  | Retrosplenial cortex | L, R | CV, SA, TA, TS |
|  | Lateral Occipital Cortex | L, R | CV, SA, TA, TS |
|  | Lateral Orbital Frontal Cortex | L, R | CV, SA, TA, TS |
|  | Lingual Gyrus | L, R | CV, SA, TA, TS |
|  | Medial Orbital Frontal Cortex | L, R | CV, SA, TA, TS |
|  | Middle Temporal Gyrus | L, R | CV, SA, TA, TS |
|  | Paracentral Lobule | L, R | CV, SA, TA, TS |
|  | Parahippocampal Gyrus | L, R | CV, SA, TA, TS |
|  | Pars Opercularis | L, R | CV, SA, TA, TS |
|  | Pars Orbitalis | L, R | CV, SA, TA, TS |
|  | Pars Triangularis | L, R | CV, SA, TA, TS |
|  | Pericalcarine Cortex | L, R | CV, SA, TA, TS |
|  | Postcentral Gyrus | L, R | CV, SA, TA, TS |
|  | Posterior Cingulate Cortex | L, R | CV, SA, TA, TS |
|  | Precentral Gyrus | L, R | CV, SA, TA, TS |
|  | Precuneus Cortex | L, R | CV, SA, TA, TS |
|  | Rostral Anterior Cingulate Cortex | L, R | CV, SA, TA, TS |
|  | Rostral Middle Frontal Gyrus | L, R | CV, SA, TA, TS |
|  | Superior Frontal Gyrus | L, R | CV, SA, TA, TS |
|  | Superior Parietal Cortex | L, R | CV, SA, TA, TS |
|  | Superior Temporal Gyrus | L, R | CV, SA, TA, TS |
|  | Supramarginal Gyrus | L, R | CV, SA, TA, TS |
|  | Temporal Pole | L, R | CV, SA, TA, TS |
|  | Transverse Temporal Cortex | L, R | CV, SA, TA, TS |
|  | Hemisphere | L, R | SA |
|  | Total Intracranial Volume | Bilateral | CV |
| Subcortical features (n = 44) | Accumbens Area | L, R | SV |
|  | Amygdala | L, R | SV |
|  | Caudate | L, R | SV |
|  | Cerebellum Cortex | L, R | SV |
|  | Cerebellum White Matter | L, R | SV |
|  | Cerebral Cortex | L, R | SV |
|  | Cerebral White Matter | L, R | SV |
|  | Choroid Plexus | L, R | SV |
|  | Hippocampus | L, R | SV |
|  | Inferior Lateral Ventricle | L, R | SV |
|  | Lateral Ventricle | L, R | SV |
|  | Pallidum | L, R | SV |
|  | Putamen | L, R | SV |
|  | Thalamus | L, R | SV |
|  | Ventricle Diencephalon | L, R | SV |
|  | Vessel | L, R | SV |
|  | Brain Stem | Bilateral | SV |
|  | Corpus Callosum Anterior | Bilateral | SV |
|  | Corpus Callosum Central | Bilateral | SV |
|  | Corpus Callosum Middle Anterior | Bilateral | SV |
|  | Corpus Callosum Middle Posterior | Bilateral | SV |
|  | Corpus Callosum Posterior | Bilateral | SV |
|  | Cerebrospinal Fluid | Bilateral | SV |
|  | Fourth Ventricle | Bilateral | SV |
|  | Non White Matter Hypointensities | Bilateral | SV |
|  | Optic Chiasm | Bilateral | SV |
|  | Third Ventricle | Bilateral | SV |
|  | White Matter Hypointensities | Bilateral | SV |

Note: CV, cortical volume; SA, cortical surface area; TA, cortical thickness average; TS, cortical thickness of standard deviation; SV, subcortical volume; L, left hemisphere; R, right hemisphere.
